# Discrete Event Simulation Models for CT Examination Queuing in West China Hospital

**DOI:** 10.1155/2016/2731675

**Published:** 2016-07-28

**Authors:** Li Luo, Hangjiang Liu, Huchang Liao, Shijun Tang, Yingkang Shi, Huili Guo

**Affiliations:** ^1^Business School, Sichuan University, Chengdu 610065, China; ^2^West China Hospital, Sichuan University, Chengdu 610041, China

## Abstract

In CT examination, the emergency patients (EPs) have highest priorities in the queuing system and thus the general patients (GPs) have to wait for a long time. This leads to a low degree of satisfaction of the whole patients. The aim of this study is to improve the patients' satisfaction by designing new queuing strategies for CT examination. We divide the EPs into urgent type and emergency type and then design two queuing strategies: one is that the urgent patients (UPs) wedge into the GPs' queue with fixed interval (fixed priority model) and the other is that the patients have dynamic priorities for queuing (dynamic priority model). Based on the data from Radiology Information Database (RID) of West China Hospital (WCH), we develop some discrete event simulation models for CT examination according to the designed strategies. We compare the performance of different strategies on the basis of the simulation results. The strategy that patients have dynamic priorities for queuing makes the waiting time of GPs decrease by 13 minutes and the degree of satisfaction increase by 40.6%. We design a more reasonable CT examination queuing strategy to decrease patients' waiting time and increase their satisfaction degrees.

## 1. Introduction

The healthcare resources in China are currently facing the pressure between demand and supply: 20% of the world population sharing no more than 3% of the world healthcare resources [1, 2]. With the national economy development and citizen's living standard improvement, people pay more and more attention to their health. The ever-increasing demand of medical service makes it tighter especially in the large-scale public hospitals [3]. Medical examinations require excellent expertise and expensive equipment with high accuracy, high input-output frequency, and high complexity. These resources are critical in hospitals because they are open to the patients from outpatient department, emergency department, and inpatient department, simultaneously [4]. Among different types of medical examinations, the CT examination has transformed from a specialized diagnostic examination to a more routinely used method [5]. According to the Assessment Manual of Hospital issued by the Ministry of Health of China (2008), there is a great demand of CT examination in China due to the high amount of population. The limited resource of hospitals for CT examination motivates the consideration of better queuing strategies to improve the performance and efficiency of CT examination.

West China Hospital (WCH) is one of the largest hospitals in China. The radiology department of WCH owns six CTs, with normal working hours of 8:00–21:00, and the machine utilization rate closes to 100%. Due to the setup time, prescan test, and others, each CT is allocated to do specific scans classified by the area of body, the function, and so on. For instance, besides the two specialized CTs (one mobile CT and the other for regular physical checkup), WCH has one CT dedicated to ordinary scans, two dedicated to enhanced scans which require oral and/or intravenous contrast, and one dedicated to enhanced scans of coronary heart and vessel. In summary, there is only one CT piece of equipment provided for ordinary scans in WCH. There are multiple sources of patients who need ordinary scans, including the outpatients, the inpatients, and the emergency patients (EPs). By contrast, the enhanced scans are mainly for checking the inpatients, with relatively smaller demands, and the queuing problem is not so urgent. Therefore, in this paper, we focus on the ordinary scans in CT examination in WCH.

Basically, according to the emergency degrees, the patients are divided into general type and emergency type for medical examination in WCH [6]. The general patients (GPs) need to make an appointment for an examination while the EPs could wedge to the queue directly before all GPs for an examination. The unlimited times of wedging to the queue from EPs have a tremendously bad influence on the GPs' satisfaction degrees. It leads the overall patient satisfaction degrees to be low and thus attracts the attention of hospital managers and academic circles.

To validate and compare the performances of different CT examination queuing strategies, modeling the queuing is a helpful means to better understand the situations of different types of patients in the queue. Discrete event simulation [7] is a type of modeling method in the field of systems engineering and it was widely used in various studies [8–12]. In the application area of healthcare, the literature on the subject is diverse. Ceglowski et al. [13] built a discrete event simulation model to identify particular bottlenecks in the important flow problem of patients admitted to hospital beds from the emergency department. Zeng et al. [14] explored various qualities of care indicators, including the length of stay, the waiting times, and the patient premature departures in a community hospital's emergency department. They determined that deploying a team nursing policy could lead to substantial improvements in the hospital system's key indicators. Jahangirian et al. [15] illustrated lessons from commerce and defense that could inform simulation applications in healthcare. Viana et al. [16] developed models to address healthcare decision-making and analysis involving Chlamydia, a sexually transmitted infection. The researchers deployed a discrete-event simulation model to analyze hospital outpatient clinic flow and a systems dynamic model to investigate the infection process in the larger population.

Many researchers attempted to combine simulation with queuing techniques and achieved practical insights and results which are highly fruitful in healthcare system [17–21]. It is found that most literatures focused on the outpatient service department, operation department, and emergency department. Joustra et al. [19] examined whether the urgent and regular patients waiting for a consultation at a radiotherapy outpatient department should be pooled or not. The practical approach indicates that the separation of queues may require less capacity to meet the waiting time performance target for urgent as well as regular patients. Aboueljinane et al. [20] proposed a discrete event simulation model implemented in the ARENA software to analyze the possible changes in the French Emergency Medical services processes that would lead to the enhanced operational efficiency for coverage performance. Mokaddis et al. [21] developed a multiclass Markovian queuing network model of patient flow in the accident and emergency department of a major hospital in the Egypt Health Service. Using a discrete-event simulation, they investigated the impact of giving priority treatment to different classes of patients and compared the resulting response time densities and moments with real data. In China, Zhang et al. [22] from Shanghai Jiao Tong University set the different weights of parameters based on the priority level of each outpatient and the proportion they took in the waiting line. Having taken the patients' waiting time and the number of patients into account, they dynamically adjusted the patients' orders in the waiting line and proposed a queuing model which dynamically adjusts itself and designs relevant calculation. They verified the effectiveness and feasibility of the model by testing in real situations. In addition, by the application of information process, automation management was achieved, which reduced the patients' waiting time and boosted the overall satisfaction rate. But the studies on the queuing problems regarding medical examination, especially CT examination, are quite few.

What is more, as for the research on medical examination, most literatures focused on how to maximize the utilization rate of medical examination equipment and minimize the cost. Rosenquist [23] pointed out that the medical examination should first focus on the factors such as the arrival status of patients, the conditions of equipment, and the number of waiting patients. By applying the method of stock management in revenue management, Green et al. [24] sought a sound solution to maximize the efficiency of medical examination equipment. Patrick et al. [25] focused on the research of the schedule of patients in certain medical examination departments with dynamic priority queuing system, which minimizes the patients' waiting time and the cost of hospital. However, in practice, the medical examination equipment usually works beyond its capacity, which results in the problem that the patients always need to wait for medical examination, and such problem is particularly urgent in China. In this study, we take the CT examination in WCH as a case. The main objective of this study is to decrease the waiting time and improve the patients' satisfaction degree by designing new queuing strategies. We develop some discrete event simulation models for the designed strategies.

## 2. Methods

### 2.1. Data and Problem Analysis

Our simulation models are based on a data set which includes 103248 CT examination records of patients collected and compiled from the RID of WCH from February 1 to July 31 in 2012, after obtaining appropriate research authorization. The day began at 8:00 AM and finished at 9:00 PM. The data of arrive time, examine time, leave time, and the types of patients was collected for each patient.

After processing and analyzing the gathered data through SPSS, we calculated the EPs' and the GPs' average waiting time (see Table 1) and also counted the distribution of GPs' waiting time (see Table 2).

Several facts can be discovered after checking Tables 1 and 2:

1. The proportion of the EPs is more than 34%, which is much higher than the reality or the medical research's hypothesis (7%~10%) [26–29]. After some interviews and investigation, we find the reason for this is that there are many patients who are not so serious but take the emergency examination because they accept the comparatively higher standard of emergency examination. For example, some endemic patients want to temporarily accept CT examination to confirm whether they are suitable for surgery or not; some nonnative patients may ask the doctor to endow them the emergency priorities in order to get the diagnose results quickly; in addition, some VIP patients always take the higher standard examination.
2. Lots of EPs wedged in the queue so the GPs have to wait for a long time. The average waiting time of the GPs is over 1 hour. Only 26.5% of them can finish the examination within 30 minutes. Over 50% of GPs need to wait for at least one hour. 16.2% of GPs even need to wait for more than 2 hours while the EPs can accept the examination within a very short time.

To solve the existing problems, the following solutions are proposed:

1. Classify the EPs according to their severity conditions. They can be further divided into the EPs and the urgent patients (UPs). EPs are under serious conditions and must be examined as soon as possible. UPs are not so serious but need to be examined as quickly as possible, and they allow a short wait. After consulting with the CT inspectors, we can find that, in the original EPs, the EPs account for 30% and the UPs occupy 70%. The EPs can occupy the highest priorities while the UPs have the dynamic priorities.
2. Design the queuing strategies. According to advice of the CT inspectors and nurses in WCH, we suppose the thresholds of waiting time for the UPs and the GPs are 20 minutes and 40 minutes, respectively. Given that patients would feel anxious when the thresholds are exceeded, two strategies can be designed: one is the UPs wedge into the GPs' queue with fixed interval and the other is the UPs have dynamic priorities for queuing. By these means, the EPs can finish their examinations within proper waiting time while the waiting time of GPs can be reduced to some extent.

### 2.2. Base Model

**Figure 1:**
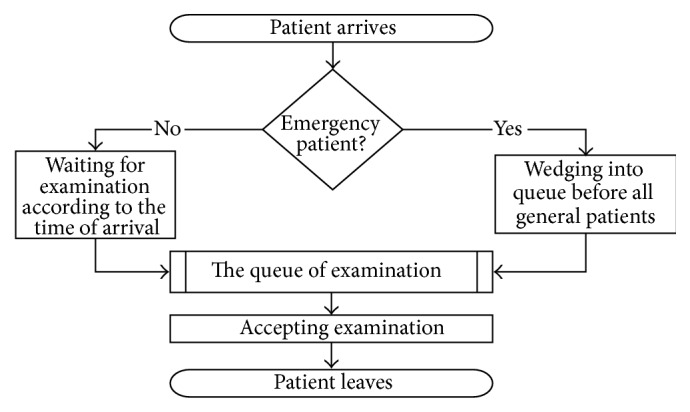
The process for patient to accept CT examination.

Our simulation models are constructed based on the base model which describes the situation of the CT examination queuing.  Figure 1 provides a summary of the process that a patient accepts the CT examination in WCH.

From Figure 1, we can find that the input parameters that the simulated model needs to include involve the patients' arrival time and the examination durations of different types of patients:

1. Patient's arrival time (min): By analyzing the data from WCH, the arrival time of GPs follows an empirical distribution. And the arrival time of EPs follows an exponential distribution with the parameter equal to 7.
2. Examination duration (min): Based on the data from WCH, the examination durations of patients follow empirical distribution. And the average examination durations of GPs and EPs are 2.1 min and 3.2 min, respectively.  Table 3 lists the examination durations of GPs and EPs.

The mainly output indicators in this paper include the satisfaction rate, the average waiting time for examination, and the daily examination quantity. The satisfaction rate is the rate of patients whose waiting time is within the thresholds of waiting time [30].

The model is built using SIMIO. After establishing the base model, we run the model under the same situation of the collected data and compare the outputs with the historical data.  Table 4 shows that the results of base model match closely with the historical data. It is agreed that the base model is effective and can reflect the actual CT examination queuing well. Therefore, it is able to evaluate the correlated indicators by simulating actual system.

## 3. Simulation Models

In this section, we design two strategies of CT examination queuing and develop the simulation models under each strategy. The first strategy is to wedge the UPs into the GPs' queue with fixed interval (fixed priority model). The second strategy is to put the UPs into the queue on the basis of dynamic priority and adjust the orders of both the UPs and the GPs based on their orders and waiting time dynamically to ensure that the GPs can finish the examination within the given time as many as possible (dynamic priority model).

### 3.1. Fixed Priority Model

The difference between the fixed priority model and the base model is that the EPs in the base model are divided into two groups, namely, the UPs and the EPs. The UPs are given priorities to wedge into the GP queue based on the status of the queue. Briefly, the priorities of GPs are always 1, the initial priorities of UPs are 2, and the initial priorities of EPs are 1.

We obtained the average examination duration of GPs which is 2.1 minutes. Meanwhile, it should also be noted that the UPs would be discontented if they are asked to wait over 20 minutes. Thus, in the simulated strategy, we stipulate that the UPs are allowed to wedge 10 GPs in the queue at most; otherwise it will be beyond the tolerance of GPs. As a result, the number of GPs who are wedged can be 0, 1, 2, 3, 4, 5, 6, 7, 8, 9, or 10. That is to say, there are 10 schemes for fixed priority model. Although the UPs can tolerate to wait, there should be an acceptable time limit. We assume that only 5% of UPs are willing to wait for over 20 minutes.

**Figure 2:**
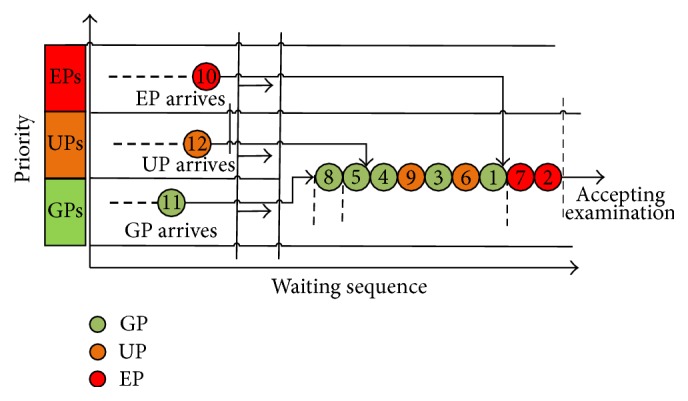
Queuing diagram of fixed priority model.

The GPs, the UPs, and the EPs have different priorities in the simulation process. The UPs enjoy the highest priorities which remain constant. The GPs have descending priorities which are subject to their arriving orders. Meanwhile, the patients' orders are determined by the First-In-First-Out (FIFO) rule. The priorities given to the UPs are determined by the situations of the waiting queue of GPs. The fixed priority model with 1 UP wedging into the queue of GPs is illustrated in Figure 2.

Assume the interval number of UPs wedging into the queue of GPs is *P*. When an UP arrives, there will be three situations:

(I) If the last patient in the examination queue is an EP, which indicates all the patients in the queue are EPs, the UP waits directly after the last EP and enjoys the same priority.
(II) If the last patient in the examination queue is an UP, the UP takes the same priority as the previous UP.
(III) If the last patient in the examination queue is a GP, then we shall try to find the nearest UP:
  (a) If there is no UP, continue to search the location of the latest EP from the end of the queue and determine his/her priority.
    (i) If there is still no EP, then count the number of GPs. If the number of GPs is more than *P*, then the priority of the new UP is the first GP's priority plus *P*; otherwise the priority of the new UP is the same as that of the last GP in the queue.
    (ii) If an EP is found, then count the total number of GPs behind the EP. If the number of GPs is more than *P*, then the priority of the new UP is the first GP's priority plus *P*. If the number of GPs is less than *P*, then the priority of the new UP is the same as that of the last GP.
  (b) If the last UP is found, then count the total number of GPs behind that UP. If the number of GPs is more than *P*, then the priority of the new UP is the UP's priority plus *P*. If the number of GPs is less than *P*, then the priority of the new UP is the same as that of the last GP.

### 3.2. Dynamic Priority Model

**Figure 3:**
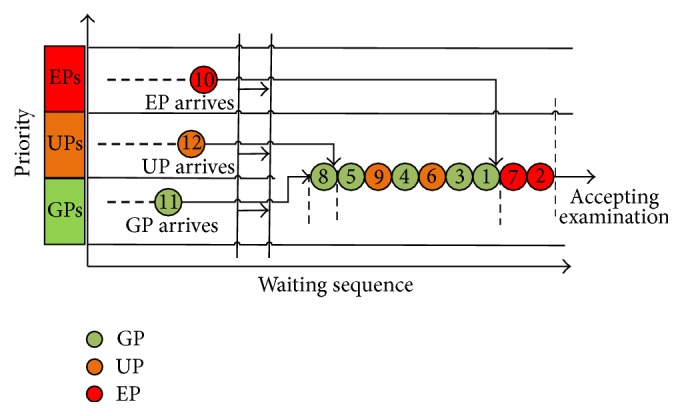
Queuing diagram of dynamic priority model.

Based on the base model, the dynamic priority model adjusts the patients' priorities (except for the EPs) according to their waiting time. It assumes that the UPs should wait shorter than the GPs before taking the examination. The priority of each type of patients is adjusted dynamically according to the arrival order, the waiting time, and the degree of emergency (shown as Figure 3). In this dynamic model, the group with the highest rank of priority (which is 1) will be able to take the examination earlier. Due to the severity of EPs, we grant them with the highest rank of priority which remains unchanged. The priority ranks of the UPs and the GPs should be increased as they wait longer. When one patient waits longer than the waiting time threshold, they will enjoy the same priority rank as that of the EPs. When they wait shorter than the waiting time threshold, their priority rank goes up as they wait longer but is lower than that of the EPs.

Assume the priorities of the GPs, UPs, and EPs are *f*_GP_, *f*_UP_, and *f*_EP  _, respectively, and the waiting times of them are *t*_GP_, *t*_UP_, and *t*_EP_, respectively. The priorities of the EPs keep unchanged as *f*_EP_ = 1. The priorities of the UPs decrease as their waiting time increases and reach to 1 when their waiting time comes to the threshold 20 minutes, shown as

(1) $$\begin{array}{c} f_{UP}=\left\{\begin{array}{cc} 1, & t_{UP}\geq 20,\\ \frac{20}{t_{UP}}\times 1, & t_{UP}<20. \end{array}\right. \end{array}$$

The priorities of GPs also decrease as the waiting time increases. Due to the fact that the conditions of GPs are not as urgent as those of the UPs, their acceptable waiting time threshold should be larger than that of the UPs. Here we set this threshold as 40 minutes, which means when the waiting time comes to 40 minutes, their priority reaches to 1, shown as

(2) $$\begin{array}{c} f_{GP}=\left\{\begin{array}{cc} 1, & t_{GP}\geq 40\\ \frac{40}{t_{GP}}\times 1, & t_{UP}<40. \end{array}\right. \end{array}$$

## 4. Results

### 4.1. Fixed Priority Model

For the fixed priority model, input the parameters and conduct every scheme for 100 times; then we can get the results of output indicators, which are shown in Table 5.

**Figure 4:**
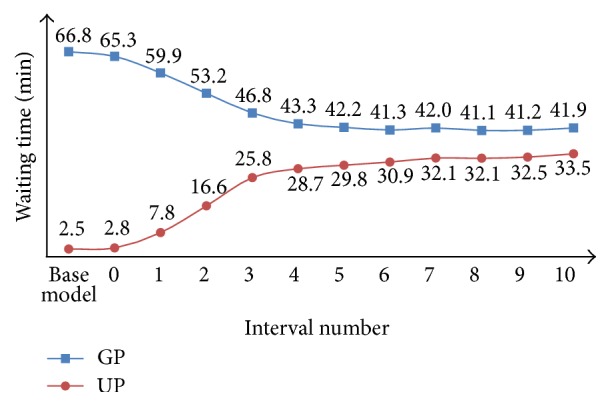
Performance comparison between the base model and the fixed priority model for GPs and UPs in terms of waiting time.

From Figure 4, we can find that the waiting time of GPs and UPs changes significantly with the increasing of *P*. The waiting time of GPs decreases and the slope of the decreasing curve becomes stable with the increasing of *P*. The waiting time stands at 41.9 minutes at last. By contrast, the waiting time of UPs increases and the slope of the waiting time curve becomes stable as well with the increasing of *P*. The waiting time comes to about 32 minutes finally. In addition, we can see that when 1 or 2 patients wait in front, that is, *P* = 1 or 2, the waiting time curve of GPs decreases sharply and the waiting time curve of UPs increases sharply. Therefore, when there are 1 or 2 patients waiting ahead, the waiting time is most favorable for both of the groups of patients.

**Figure 5:**
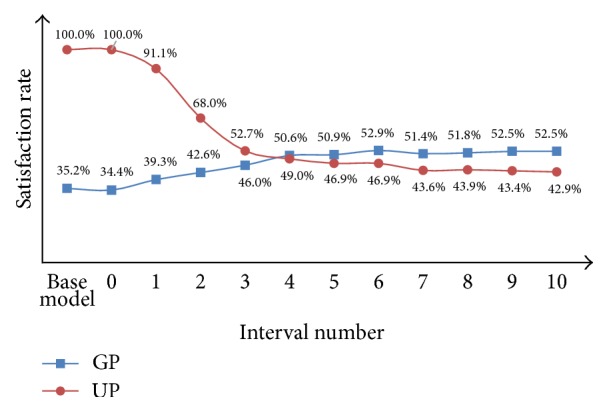
Performance comparison between the base model and the fixed priority model for GPs and UPs in terms of satisfaction rate.

It is noted that the satisfaction rate is related to the proportion of patients who wait for the examination within an acceptable period of time. It can be seen from Figure 5 that, with the increasing of *P*, the satisfaction rate of GPs increases continually while the satisfaction rate of UPs decreases accordingly. The satisfaction rate of GPs changes gently while that of the UPs changes sharply. In particular, the satisfaction rate of UPs goes down directly from 91.1% (*P* = 1) to 68.0% (*P* = 2). In summary, it is appropriate to keep the fixed interval number of patient at 1.

### 4.2. Dynamic Priority Model

**Figure 6:**
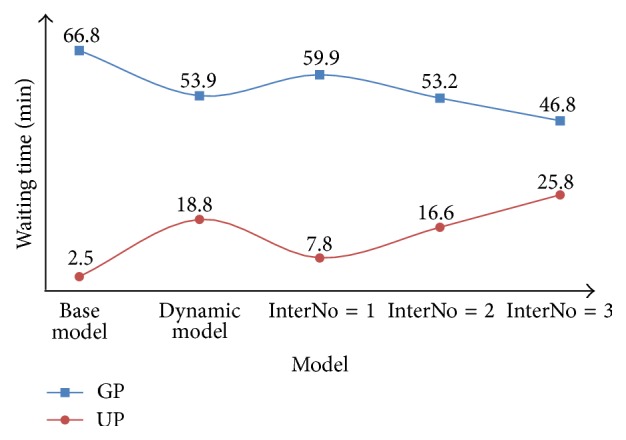
Comparison between the base model, the dynamic priority model, and the fixed priority model for GPs and UPs in terms of waiting time.

**Figure 7:**
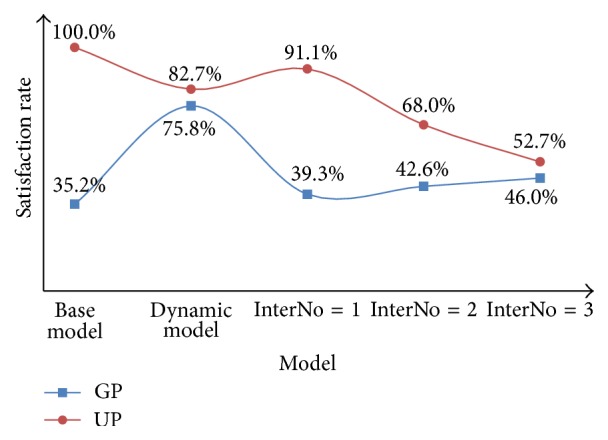
Comparison between the base model, the dynamic priority model, and the fixed priority model for GPs and UPs in terms of satisfaction rate.

For the dynamic priority model, input the parameters and conduct the scheme for 100 times. We combine the results of this model with the results of the base model, which are shown in Table 6 and Figures 6 and 7.

From the results of the fixed priority model, we can see that the best strategy is to keep *P* = 1, which can make the waiting time of GPs be shorter by 7 minutes and maintain the waiting time of UPs within 8 minutes. Meanwhile, the satisfaction rate of UPs can be kept at 91.1% while the satisfaction of GPs can be raised by 4.0%.

However, from the results of the dynamic priority model, we can find that the waiting time of GPs is shortened by 13 minutes, and the waiting time of UPs is 18.8 minutes which is restricted within 20 minutes. Furthermore, although the satisfaction rate of UPs drops to 87.2%, the satisfaction rate of GPs rises from 35.2% to 75.8%, which makes the overall satisfaction rate rise by 40.6%.

Therefore, the dynamic priority strategy is superior to the fixed priority strategy.

## 5. Discussions

Our research is motivated by hospital operation in Chinese environment and the idea is to improve patients' satisfaction through redesigning queuing strategy. Mostly, improving patients' satisfaction is done by maximizing the utilization rate of medical examination equipment. But when the demand exceeds supply, the medical machine utilization rate closes to 100% and there is limited space for further improving. In such situation, more approaches such as designing queuing strategy can be considered.

The queuing strategy is an important factor that may significantly affect the waiting time for service. McQuarrie [31] showed that it is possible, when resource utilization rate is high, to minimize the waiting time by giving priorities to clients who require shorter service time. This rule is a form of the shortest processing time rule that is known to minimize waiting time. But it is found infrequently in practice due to the perceived unfairness and the difficulty of estimating service time accurately. Au-Yeung et al. [32] developed a multiclass Markovian queuing network model of patient flow in the accident and emergency department of a major London hospital. They experimented with different patient handling priority schemes and compared the resulting response time moments and densities with real patient timing data. Zhang et al. [22] set the different weights of parameters based on the priority level of each outpatient and the proportion they took and proposed a queuing model which dynamically adjusts the patients' orders in the waiting line. They showed that it can reduce the patients' waiting time and boost the overall satisfaction rate. Creemers and Lambrecht [33] concentrated on service outages and developed new expressions to assess their impact on waiting lists and delays. Using data obtained from a Belgian hospital, the expressions were evaluated through a number of queuing models. So redesigning queuing strategy of CT examination is feasible to improve the patient's waiting time and satisfaction.

In this paper, based on the data from WCH, we divide the EPs into urgent type and emergency type according to their severity conditions. Then we create some discrete event simulation models of CT examination with two different queuing strategies. One strategy is to wedge the UPs into the GPs' queue with fixed intervals (which established 11 different fixed priority models). Another strategy is to put the UPs into the queue on the basis of dynamic priorities and adjust the orders of both the UPs and the GPs based on their orders and waiting time dynamically to ensure that the GPs can finish the examination within the given time as many as possible. We validate and compare the performance of different CT examination queuing strategies through the simulation models. The result of Table 6 shows that both strategies are able to reduce the average waiting time and improve satisfaction rate of patients in CT examination. It is suggested that using specific queuing strategy to manage waiting periods in CT examination can make the average waiting time of patients shorter and improve the patients' satisfaction rate. In addition, the result also shows that we can improve the quality of medical service through the process improvement. This study demonstrates that the discrete event simulation approach is a potent tool for process improvement in healthcare systems. It can help researchers and healthcare managers to save implementation costs and reduce time consumption.

## 6. Conclusions and Further Research

Due to the situation that the current medical service fails to meet the demand and the optimization of the appointment system cannot boost capacity further, this paper has analyzed the current characteristics of medical service and studied the queuing strategies for CT examination to reduce the patients' waiting time and raise the satisfaction rate of patients. Through the investigation in a large-scale general hospital, we have divided the patients who wait for the CT examination into three groups, including the GPs, the UPs, and the EPs. By the discrete event simulation and SIMIO software, we have made the priority rank of GPs be unchanged and then adjusted the priority rank of UPs at fixed intervals which cover 11 simulation strategies with the intervals of patients varying from 0 to 10. We have found that the result is optimal when the interval number *P* = 1 or 2. Then, we have established a model by dynamic priority strategy which adjusts the priority ranks of GPs and UPs dynamically. We have found that the dynamic priority strategy can raise the satisfaction rate of GPs to a great extent, get better indicators of assessment than the fixed priority strategy, cut the average waiting time of GPs by 13 minutes, and raise the satisfaction rate of GP by 40.6%.

The research of this paper is based on real data, and the research method can be applied to the majority of other hospitals. Hospital managers can draw on the results of this paper to solve the appointment problem of patients.

The future study can further consider the following two aspects:

- The process simulation of other medical examination departments is the first. Typical as CT examination is, there is a big difference between different types of pathological examination processes [34]. Thus, the dynamic priority strategy can be applied to other departments.
- Dynamic priority queuing based on multiple examination equipment working in synergy is the second. This paper only focused on examination problems with one set of CT equipment. For medical examination conducted by multiple equipment, it is worth further studying how to optimize synergetically and how to adjust based on dynamic priority strategy.

## Figures and Tables

**Table 1:** The proportion and average waiting time of patients.

Patient types	Proportion	Average waiting time (min)
Overall patients	100%	44.8
EPs	34.2%	2.5
GPs	65.8%	66.8

**Table 2:** The distribution of GPs' waiting time.

Patient types	Waiting time (min)	Proportion
GPs	<30	26.5%
	30~60	21.6%
	60~90	22.1%
	90~120	13.6%
	>120	16.2%

**Table 3:** The examination durations of GPs and EPs.

Examination durations (min)	GPs		EPs	
	Frequency	Cumulative frequency	Frequency	Cumulative frequency
1	11.1%	11.1%	2.4%	2.4%
2	57.9%	69.0%	34.1%	36.5%
3	24.9%	93.9%	32.0%	68.5%
4	6.1%	100.0%	15.5%	84.0%
5	—	—	7.5%	91.5%
6	—	—	6.1%	97.6%
7	—	—	2.4%	100.0%

**Table 4:** Results of base model comparing with the historical data.

Evaluation indicator	Outputs		
	Historical data	Base model	95% confidence interval of mean value
Daily examination quantity	245	246.5	(234.2, 258.8)
The average waiting time of GPs	66.8	64.0	(60.8, 67.2)
The average waiting time of EPs	2.5	2.4	(2.3, 2.5)
The satisfaction rate of GPs	—	35.2%	(38.3%, 42.3%)

*Note.* In practice, no one measured the satisfaction rate of GPs, so we cannot compare the result obtained from the base model with the historical data.

**Table 5:** Results comparison between the base model and the fixed priority model.

Evaluation indicators	Base model	Fixed priority model										
		Interval number										
		0	1	2	3	4	5	6	7	8	9	10
Waiting time (min)												
(i) EPs	2.5	1.5	1.6	1.5	1.5	1.5	1.6	1.5	1.5	1.5	1.6	1.5
(ii) GPs	66.8	65.3	59.9	53.2	46.8	43.3	42.2	41.3	42.0	41.1	41.2	41.9
(iii) UPs	2.5	2.8	7.8	16.6	25.8	28.7	29.8	30.9	32.1	32.1	32.5	33.5
(iv) The average of total waiting time	36.5	37.1	35.5	34.9	34.0	32.9	32.7	32.5	33.2	32.7	32.9	33.6
Examination rate (%) in temporary threshold												
(i) The satisfaction rate of GPs	35.2%	34.4%	39.3%	42.6%	46.0%	50.6%	50.9%	52.9%	51.4%	51.8%	52.5%	52.5%
(ii) The satisfaction rate of UPs	100%	99.7%	91.1%	68.0%	52.7%	49.0%	46.9%	46.9%	43.6%	43.9%	43.4%	42.9%
Daily examination quantity	246.5	247.7	246.1	246.6	247.5	245.5	246.7	245.7	247.1	246.9	246.4	246.8

**Table 6:** Comparison between the base model, the fixed priority model, and the dynamic priority model.

Evaluation indicators	Base model	Fixed priority model			Dynamic priority model
		Interval number (InterNo)			
		1	2	3	
Waiting time (min)					
(i) EPs	2.5	1.6	1.5	1.5	1.6
(ii) GPs	66.8	59.9	53.2	46.8	53.9
(iii) UPs	2.5	7.8	16.6	25.8	18.8
(iv) The average of total waiting time	36.5	35.5	34.9	34.0	35.6
Examination rate (%) within the maximum waiting time					
(i) The satisfaction rate of GPs	35.2%	39.3%	42.6%	46.0%	75.8%
(ii) The satisfaction rate of UPs	100%	91.1%	68.0%	52.7%	87.2%
Daily examination quantity	246.5	246.1	246.6	247.5	246.6
